# Evaluation of the immune factors in the tumor environment before and after the treatment of cetuximab combined with chemotherapy

**DOI:** 10.1186/1477-7819-11-226

**Published:** 2013-09-13

**Authors:** Yanyun Zhu, Jie Li, Fangfang Jing, Tiefeng Ji, Xiaoqin Guo, Junlan Yang, Shunchang Jiao

**Affiliations:** 1 Department of Medical Oncology, Chinese PLA General Hospital, 28 Fuxing Road, Haidian District, Beijing 100853, China; 2 Department of Pathology, Chinese PLA General Hospital, 28 Fuxing Road, Haidian District, Beijing 100853, China; 3 The First Out-patient Department, Bureau of Management and Social Security, Headquarters of the General Logistics Department of PLA, 22 Fuxing Road, Haidian District, Beijing 100842, China

**Keywords:** Cetuximab, Chemotherapy, Tumor environment, Immune state, Clinical efficacy

## Abstract

**Background:**

The effect of chemotherapy combined with monoclonal antibodies (mAbs) on the immune state of the tumor environment remains unclear and controversial. The aim of this study is to examine the effect of chemotherapy combined with cetuximab (C225, an anti-EGFR mAb) on the immune state of tumor environment, and the correlation of that effect and the clinical efficacy.

**Methods:**

Twelve patients with colorectal cancer, who received the treatment of chemotherapy combined with C225, were enrolled in this study. The tumor specimen of the primary colorectal cancer before and after treatment was obtained. The expression of a series of immune factors (TGF-β1, CD8, IL-2, TNF-α, and VEGF) was measured by immunochemistry. The expression of these immune factors before and after treatment was compared by the Wilcoxon signed-rank test. The correlation of the change of immune parameter expression after treatment and clinical efficacy was examined by chi-square tests. The correlation of the expression of immune factors, clinical efficacy, and treatment number was examined by the Spearman’s correlation analysis.

**Results:**

There was no significant difference between the expression of TGF-β1 before and after the treatment (*P* >0.05). The change of TGF-β1 expression after treatment significantly correlated negatively with clinical efficacy (*P* = 0.05). As for CD8, IL-2, VEGF, and TNF-α, there were no significant differences between the expression before and after the treatment (*P* >0.05), and the change of expression after treatment also did not correlate significantly with clinical efficacy (*P* >0.05). The change of IL-2 expression after treatment significantly correlated negatively with treatment number (correlation coefficient = -0.585, *P* = 0.046). The change of TGF-β1 expression after treatment significantly correlated negatively with clinical efficacy (correlation coefficient = -0.684, *P* = 0.014). Before treatment, the expression of TNF-α significantly correlated positively with the expression of IL-2 (correlation coefficient = 0.629, *P* = 0.028). After treatment, the expression of TGF-β1 significantly correlated negatively with the expression of CD8 (correlation coefficient = -0.664, *P* = 0.019).

**Conclusions:**

These results suggested that, in the tumor environment, the change of immune factors after treatment of cetuximab combined with chemotherapy may be associated with clinical efficacy.

## Background

The mechanism of antitumor immunity is very complex, involving with various immune cells and their secretory cytokines [1]. It is well known that antitumor immunity is mainly mediated by cellular immunology, whereas humoral immunity (denoting immune responses that are mediated by antibodies) does not play an important role in antitumor immunity [2]. In some cases, humoral immunity plays a positive role in antitumor immunity [3]. However, humoral immunity can impair specific immune responses that recognize and attack tumor cells in some cases, resulting in the proliferation and invasion of tumor cells [1]. It is reported that humoral immunity can also induce immune response, and can enhance the effect of immune response to tumor specific antigens, when combined with other therapeutic approaches [2]. Thus, the immunomodulatory effect of humoral immunity (antibodies) remains controversial.

Cetuximab (Erbitux, C225) is an anti-EGFR (epidermal growth factor receptor) antibody used for the treatment of metastatic colorectal cancer and head and neck cancer [4]. Cetuximab is a chimeric (mouse/human) monoclonal antibody given by intravenous infusion that is manufactured and distributed by the drug companies Bristol-Myers Squibb and Eli Lilly and Company. When combined with radiotherapy and chemotherapy, cetuximab has shown synergistic and potential antitumor activity in many cancers [5]. The antitumor mechanism of cetuximab is complex, consisting of blockade of the cell signaling that contributes to cell proliferation, antibody-dependent cellular cytotoxicity (ADCC), and complement-dependent cytotoxicity (CDC) [6]. At present, there have been few reports about the immunomodulatory effect of antibodies, except ADCC and CDC [7]. On the contrary, there have been many studies about the immunomodulatory effects (immunosuppressive effects) of chemotherapy [8]. Chemotherapy is currently believed to inhibit the cellular immunity, reflecting by the observed situation in clinic that the more the cycle of chemotherapy is, the more severe the immunosuppressive effect is [9]. Slovin showed that the immune state could return to normal levels in patients whose lesion relieved after chemotherapy, whereas the immune state could not return to normal levels in patients whose lesion did not relieve after chemotherapy [8]. Recently, several reports indicated that the original immune state is closely associated with the prognosis of cancer patients [10,11]. However, the effect of chemotherapy combined with monoclonal antibodies (mAbs) on the immune state of the tumor environment remains unclear and controversial. Furthermore, the effect of the immune state on the clinical efficacy of cancer patients also remains unclear.

In this study, 12 patients with colorectal cancer, who received the treatment of chemotherapy combined with C225, were enrolled in this study. The tumor specimen of the primary colorectal cancer before and after treatment was obtained. The expression of a series of immune factors (TGF-β1, CD8, IL-2, TNF-α, and VEGF) was measured by immunochemistry. The aim of this study is to examine the effect of chemotherapy combined with cetuximab on the immune state of the tumor environment, and the correlation of that effect and clinical efficacy.

## Methods

### Patients and specimen

Twelve patients (11 men and 1 woman) with colorectal cancer, who received the treatment of chemotherapy combined with C225 in the Department of Medical Oncology of Chinese PLA General Hospital (Beijing, China) during the 2-year period from December 2010 to June 2012, were enrolled in this study. The age of patients ranged from 21 to 60 years, with a mean age of 47.5 years. All the patients had an Eastern Cooperative Oncology Group (ECOG) performance status of 0-1. The general clinicopathological characteristics of patients were shown in Table 1.

**Table 1:** **General and clinicopathological characteristics of patients (*n *= 12)**

**Patient no.**	**Sex**	**Diagnosis**	**Regimen**	**Treatment number**	**Clinical efficacy**
1	Male	Colon cancer metastases to liver	C225 + FOLFIRI	3	PD
2	Male	Rectal cancer metastases to liver	C225 + CPT-11	3	PD
3	Male	Rectal cancer metastases to liver	C225 + FOLFOX	2	PD
4	Male	Rectal cancer metastases to LN	C225 + FOLFIRI	3	PD
5	Male	Colon cancer metastases to liver	C225 + XELOX	4	SD
6	Male	Rectal cancer metastases to liver	C225 + CPT-11	18	PR
7	Male	Rectal cancer metastases to liver	C225 + FOLFOX	5	PR
8	Male	Rectal cancer metastases to liver	C225 + FOLFOX	1	PR
9	Male	Colon cancer metastases to liver	C225 + FOLFOX	12	PR
10	Male	Colon cancer metastases to liver	C225 + FOLFOX	12	PR
11	Male	Colon cancer metastases to liver	C225 + FOLFIRI	3	PR
12	Female	Colon cancer metastases to liver	C225 + FOLFIRI	3	PR

CPT-11, Irinotecan; FOLFIRI, Irinotecan + 5-fluorouracil + calcium folinate; FOLFOX, oxaliplatin + 5-fluorouracil + calcium folinate; LN, lymph nodes; PD, Progressive Disease; PR, Partial Response; SD, Stable Disease; XELOX, oxaliplatin + capecitabine.

The criteria for inclusion in this study were: age ≥18 years; ECOG performance status ≤2; tumors were demonstrated as colorectal cancer by pathology, and did not receive the therapy of mAbs; at least one measurable tumor lesion, demonstrated by CT or MRI; an expected surviving period ≥3 months; routine blood tests performed 0-3 days before chemotherapy (absolute neutrophil ≥1.5 × 10^9^/L, platelets ≥100 × 10^9^/L, and hemoglobin ≥10 g/dL); hepatic function test (aspartate aminotransaminase and alanine aminotransferase should be <1.5-fold of the upper limit of normal values, and they should be <2.5-fold of the upper limit of normal values in patients with known hepatic metastases); renal function test (a calculated creatinine clearance rate was <45 mL/min).

The criteria for exclusion in this study were: brain metastasis; receiving the therapy of mAbs; local therapy in the tumor lesion; severe allergic reactions; no measurable tumor lesions; an expected survival period <3 months.

This study was conducted according to the International Conference on Harmonisation (ICH) Good Clinical Practice guidelines, including obtaining written informed consent from all patients.

### Experimental procedure

Twelve patients with colorectal cancer, who received the treatment of chemotherapy combined with C225, were enrolled in this study. The tumor specimen of the primary colorectal cancer before and after treatment was obtained by surgery or puncture. Informed consent was obtained from all patients. After fixation by 10% formaldehyde, the tumor specimen was embedded in paraffin and cut into 4-μm serial sections.

### Immunohistochemistry

The expression of TGF-β1, CD8, IL-2, TNF-α, and VEGF was detected by an immunohistochemistry StreptAvidin-Biotin Complex (SABC) method. Briefly, the sections were deparaffinized, hydrated to water, and incubated with 3% H_2_O_2_ for 30 min at room temperature to eliminate endogenous peroxidase. After rinsing with distilled water and immersion in phosphate buffered saline (PBS), the sections were submitted to antigen retrieval by a microwave oven in citrate buffer (pH 6.0). Ten percent of goat serum was used to eliminate non-specific staining. The sections were incubated with the primary antibody overnight at 4°C and for an additional 45 min at 37°C. Primary antibodies consist of four antibodies: goat anti-human TGF-β1, CD8, IL-2, TGF-β1, or VEGF mAbs (Zhongshan Goldenbridge Biotechnology Co., Beijing, China). The sections were then incubated with a biotinylated secondary antibody (Zhongshan Goldenbridge Biotechnology Co., Beijing, China) for 20 min at 37°C and then horseradish peroxidase (HRP)-labeled SABC for 15 min before addition of 3,3’-diaminobenzidine (DAB). Finally, the stained slides were counterstained with Hematoxylin and Eosin (HE), dehydrated, made transparent, and sealed. In the negative controls in which the primary antibody was omitted, there was no staining. Evaluation of the staining results was according to the traditional semi-quantitative grading method and judgments were independently made by two experienced pathologists through a single-blind method. According to Beesley’s immune grading method, 10 fields were randomly chosen at a ×400 magnification. For TGF-β1, CD8, IL-2, TNF-α, and VEGF, grading was determined according to the percentage of positive cell and staining intensity: a percentage of positive cells <5% or a light yellow staining was considered as (-); a percentage of positive cells between approximately 5% and 25% with brown yellow granules was considered as (+); a percentage of positive cells between approximately 26% and 50% with brown yellow granules was considered as (++); and a percentage of positive cells >55% with dark brown granules was considered as (+++).

### Response Evaluation Criteria in Solid Tumors (RECIST)

The clinical evaluation of the patients who all had measurable target lesions was performed according to the RECIST proposed by NCI and et al. [12], which was described as following: Complete Response (CR): disappearance of all target lesions; Partial Response (PR): at least a 30% decrease in the sum of the longest diameter (LD) of target lesions, taking as reference the baseline sum LD; Stable Disease (SD): neither sufficient shrinkage to qualify for PR nor sufficient increase to qualify for PD, taking as reference the smallest sum LD since the treatment started; Progressive Disease (PD): at least a 20% increase in the sum of the LD of target lesions, taking as reference the smallest sum LD recorded since the treatment started or the appearance of one or more new lesions.

### Statistical analysis

All data in this study were processed using SPSS 13.0 software. The data were pretreated as follows: the staining intensity (-), (+), (++), and (+++) were transformed to 0, 1, 2, and 3, respectively. In the correlation analysis, PD was transformed to 1, SD was transformed to 2, and PR and CR were transformed to 3. In other analyses, PD, SD, PR, and CR were transformed to two categorical variables (invalid (defined as 0, including PD) and clinically beneficial (defined as 1, including SD, PR and CR)). For group comparisons, unordered categorical variables were compared using chi-square (χ^2^) test or Fisher’s exact test. The expression of these immune factors before and after treatment was compared by the Wilcoxon signed-rank test. The Spearman correlation was used to measure the linear relationship between two datasets. A *P* value ≤0.05 was considered statistically significant.

## Results

The general and clinicopathological characteristics of the 12 patients were shown in Table 1. As shown in Table 2, six of 12 patients (50%) showed increase in the expression of TGF-β after treatment, five of 12 patients (42%) showed no change (stable), and only one patient (8%) showed decrease. In the six patients who showed increase in the expression of TGF-β after treatment, four showed PD and two showed PR. In the five patients who showed no change, four showed PR and one showed SD. After statistical analysis (Table 3), there was no significant difference between the expression of TGF-β1 before and after the treatment (Wilcoxon signed-rank tests, *P* >0.05). The change of TGF-β1 expression after treatment significantly correlated negatively with clinical efficacy (Chi-square tests, χ^2^ = 6.000, *P* = 0.05). One hundred percent (1/1) of the patients were clinically beneficial when the expression of TGF-β1 decreased, whereas 33.33% (2/6) of the patients were clinically beneficial when the expression of TGF-β1 increased.

**Table 2:** The expression of TGF-β1 before and after treatment

**Patient no.**	**Before treatment**	**After treatment**	**Change of expression**	**Treatment number**	**Clinical efficacy**
1	++	+++	Increase	3	PD
2	+	++	Increase	3	PD
3	+	++	Increase	2	PD
4	+	++	Increase	3	PD
5	++	++	Stable	4	SD
6	+++	+++	Stable	18	PR
7	+++	-	Decrease	5	PR
8	-	+	Increase	1	PR
9	++	++	Stable	12	PR
10	+	+++	Increase	12	PR
11	+++	+++	Stable	3	PR
12	+	+	Stable	3	PR

**Table 3:** The correlation of the change of TGF-β1 expression before and after treatment with clinical efficacy

**Change of expression**	**Clinical efficacy**		**Summary**
	**Invalid**	**Clinically beneficial**	
Decrease	0	1	1
Stable	0	5	5
Increase	4	2	6
Summary	4	8	12

PD, SD, PR and CR were transformed to two categorical variables (invalid (defined as 0, including PD) and clinically beneficial (defined as 1, including SD, PR, and CR)).

The number of patients was calculated.

As for CD8, IL-2, VEGF, and TNF-α, there were no significant differences between the expression of these immune factors before and after the treatment (Wilcoxon signed-rank tests, *P* >0.05, data not shown). Furthermore, the change of immune parameter expression after treatment did not significantly correlate with clinical efficacy (Chi-square tests, *P* >0.05, data not shown).

The correlation of the change of immune parameter expression after treatment (CD8, IL-2, VEGF, TNF-α, and TGF-β), clinical efficacy and treatment number was examined by the Spearman’s correlation analysis (Tables 4, 5, 6). The change of IL-2 expression after treatment significantly correlated negatively with treatment number (correlation coefficient = -0.585, *P* = 0.046). The change of TGF-β1 expression after treatment significantly correlated negatively with clinical efficacy (correlation coefficient = -0.684, *P* = 0.014). Before treatment, the expression of TNF-α significantly correlated positively with the expression of IL-2 (correlation coefficient = 0.629, *P* = 0.028). After treatment, the expression of TGF-β1 significantly correlated negatively with the expression of CD8 (correlation coefficient = -0.664, *P* = 0.019). As for CD8, VEGF, and TNF-α, the correlation of the change of immune parameter expression after treatment (CD8, IL-2, VEGF, TNF-α, and TGF-β), clinical efficacy and treatment number is not significant (*P* >0.05).

**Table 4:** The correlation of change of expression of immune factors, clinical efficacy, and treatment number was examined by the Spearman’s correlation analysis

		**Treatment number**	**Clinical efficacy**	**CD8**	**IL-2**	**VEGF**	**TNF-α**
Clinical efficacy	Correlation coefficient	0.479					
	*P* value	0.115					
	Sample size	12					
CD8	Correlation coefficient	0.027	−0.250				
	*P* value	0.935	0.433				
	Sample size	12	12				
IL-2	Correlation coefficient	−0.585	−0.120	−0.239			
	*P* value	0.046^a^	0.711	0.454			
	Sample size	12	12	12			
VEGF	Correlation coefficient	0.171	0.000	−0.167	−0.080		
	*P* value	0.594	1.000	0.605	0.806		
	Sample size	12	12	12	12		
TNF-α	Correlation coefficient	0.048	−0.112	0.391	0.000	0.050	
	*P* value	0.883	0.729	0.208	1.000	0.878	
	Sample size	12	12	12	12	12	
TGF-β	Correlation coefficient	−0.527	−0.684	−0.114	0.218	0.184	0.361
	*P* value	0.078	0.014^a^	0.724	0.496	0.568	0.249
	Sample size	12	12	12	12	12	12

^a^*P* <0.05.

**Table 5:** The correlation of expression of immune factors and clinical efficacy before treatment was examined by the Spearman’s correlation analysis

		**Clinical efficacy**	**CD8**	**IL-2**	**VEGF**	**TNF-α**
CD8	Correlation coefficient	−0.116				
	*P* value	0.719				
	Sample size	12				
IL-2	Correlation coefficient	0.093	0.117			
	*P* value	0.774	0.717			
	Sample size	12	12			
VEGF	Correlation coefficient	0.090	0.213	−0.265		
	*P* value	0.781	0.507	0.405		
	Sample size	12	12	12		
TNF-α	Correlation coefficient	0.131	0.055	0.629	0.130	
	*P* value	0.684	0.866	0.028^a^	0.688	
	Sample size	12	12	12	12	
TGF-β	Correlation coefficient	0.277	−0.413	0.312	−0.519	0.257
	*P* value	0.383	0.182	0.323	0.084	0.420
	Sample size	12	12	12	12	12

^a^*P* <0.05.

**Table 6:** The correlation of the expression of immune factors after treatment was examined by the Spearman’s correlation analysis

		**CD8**	**IL-2**	**VEGF**	**TNF-α**
IL-2	Correlation coefficient	−0.138			
	*P* value	0.669			
	Sample size	12			
VEGF	Correlation coefficient	0.173	0.252		
	*P* value	0.592	0.429		
	Sample size	12	12		
TNF-α	Correlation coefficient	0.270	0.004	0.153	
	*P* value	0.396	0.989	0.634	
	Sample size	12	12	12	
TGF-β	Correlation coefficient	−0.664^a^	0.074	−0.167	0.113
	*P* value	0.019	0.820	0.604	0.725
	Sample size	12	12	12	12

^a^*P* <0.05.

## Discussion

The effect of chemotherapy combined with monoclonal antibodies (mAbs) on the immune state of the tumor environment remains unclear and controversial. In this study, we examined the effect of chemotherapy combined with cetuximab on the immune state of the tumor environment, and the correlation of that effect and the clinical efficacy. The results showed that in the tumor environment, the change of immune factors before and after the treatment of cetuximab combined with chemotherapy may be associated with clinical efficacy and treatment number.

TGF-β, a potent immunosuppressive cytokine that can inhibit the immune system, can promote the development, invasion and metastasis of tumors [13]. TGF-β is overexpressed in various cancers [14]. In the initial stage of tumor, TGF-β can inhibit tumor proliferation, whereas TGF-β can promote the development, invasion, and metastasis of tumors at the middle or late stage of tumors [15]. It is generally accepted that inhibition of TGF-β play a positive role in promoting the antitumor immunity [16]. In this study, the change of TGF-β1 expression after treatment significantly correlated negatively with clinical efficacy. One hundred percent (1/1) of the patients were clinically beneficial when the expression of TGF-β1 decreased, whereas 33.33% (2/6) of the patients were clinically beneficial when the expression of TGF-β1 increased. These results suggested that TGF-β1 plays a negative role in the clinical efficacy of patients after the treatment of cetuximab combined with chemotherapy. We speculate that decreased expression of TGF-β1 may inhibit the immunosuppressive state of the patients and promote the antitumor immunity, whereas increased expression of TGF-β1 possesses the opposite effect. Thus, inhibition of TGF-β1 may enhance the therapeutic efficacy of treatment of cetuximab combined with chemotherapy. Our results were consistent with previous reports that TGF-β antibody or inhibitors could inhibit the invasion and metastasis of tumors [16].

CD8 is the distinctive marker of cytotoxic T cells, which are considered to be the main effecter cells in antitumor immunity [17]. IL-2, also called T cell stimulating cytokine, is secreted by T cells. IL-2 is the main cytokine that induces the proliferation of T cells [18]. Also, IL-2 can activate other killer cells and plays an important role in antitumor immunity [19]. In this study, there were no significant differences between the expression of CD8 and IL-8 before and after the treatment, indicating that the treatment of cetuximab combined with chemotherapy did not significantly affect the expression of CD8 and IL-8. Our results are inconsistent with previous reports that chemotherapy is currently believed to inhibit the cellular immunity [9].

TNF-α is produced by monocytes and macrophages, and can inhibit the proliferation of tumor cells. There were no significant differences between the expression of TNF-α before and after the treatment. Furthermore, the change of TNF-α expression after treatment did not significantly correlate with clinical efficacy. The same results were obtained in VEGF expression. These results indicated that the expression of TNF-α and VEGF might not be significantly affected by the treatment of cetuximab combined with chemotherapy, and the change of TNF-α and VEGF expression after treatment might not significantly affect the clinical efficacy.

It is reported that cytokines have extensive interaction and could be regulated by each other [18]. In this study, the expression of TNF-α significantly correlated positively with the expression of IL-2 before treatment, indicating that TNF-α and IL-2 might promote the expression of each other and additive antitumor activity was achieved. It has to be noted that, the expression of TGF-β1 significantly correlated negatively with the expression of CD8 after treatment. The reason may be that TGF-β1 could inhibit the proliferation of CD8-positive T cells, which is consistent with previous reports [20].

There are several limitations in this study. First, the sample size of this study is small (due to the difficult achievement of tumor specimen before and after treatment). Second, the control groups of a single cetuximab or chemotherapy lacks. In our further studies, we will enlarge the sample size of this study and add the control groups, to obtain more accurate conclusions.

## Conclusions

In summary, our study suggested that, in the tumor environment, the change of immune factors before and after the treatment of cetuximab combined with chemotherapy may be associated with clinical efficacy.

## Competing interests

The authors declare that they have no competing interests.

## Authors’ contributions

All authors have contributed substantially to the study. YZ, JL, FJ, and TJ contributed to the design of the study, to the recruitment of patients, to the analysis of data, and to the writing of manuscript. SJ contributed to the conception and design of the study. XG and JY gave contributions in the recruitment of patients. All authors read and approved the final manuscript.
